# Multiple functions of MLKL in liver fibrosis, from necroptosis to hepatic stellate cell activation

**DOI:** 10.7150/thno.76902

**Published:** 2022-07-25

**Authors:** Valeria Pistorio, Chantal Housset, Jérémie Gautheron

**Affiliations:** 1 Department of Chemical Sciences, University of Naples Federico II, Naples, Italy; 2 Sorbonne Université, Inserm, Centre de Recherche Saint-Antoine (CRSA), Paris, France; 3 Institute of Cardiometabolism and Nutrition (ICAN), Paris, France; 4 Assistance Publique-Hôpitaux de Paris (AP-HP), Saint-Antoine Hospital, Department of Hepatology, Reference Center for Inflammatory Biliary Diseases and Autoimmune Hepatitis (CMR MIVB-H), Paris, France

Hepatic fibrosis is the typical result of an exuberant wound healing reaction occurring after repeated or prolonged tissue injury 1. It is characterized by either an accumulation of extracellular matrix and its defective degradation, or both. Alanine aminotransaminase (ALT) and aspartate aminotransferase (AST) are indicators of hepatocellular injury 2. They are increased in virtually all chronic liver diseases, including non-alcoholic steatohepatitis (NASH), viral hepatitis, autoimmune disorders, and cholestatic liver diseases 1, 2. Hepatocellular injury induces an activation of Kupffer cells (KCs), the resident macrophages of the liver and a major source of inflammatory mediators, including cytokines, nitric oxide, chemokines, or lysosomal and proteolytic enzymes, which in turn exacerbate cytotoxicity 3. Thus, KCs play a pivotal role in hepatocellular injury 3. They promote the activation of hepatic stellate cells (HSCs) and their switch from a quiescent, retinoic acid storing phenotype to a myofibroblast-like phenotype, resulting in increased production of collagens types I and III 4. This excessive deposition of extracellular matrix in the subendothelial space of Disse disrupts the normal architecture of the hepatic lobule, and can lead to cirrhosis, liver failure, and portal hypertension, requiring liver transplantation 4. Hepatocyte cell death is now considered as the primary trigger for the persistent leukocyte infiltration and inflammation that fuels the fibrogenic process 5. Therefore, understanding and ultimately being able to control hepatic cell death in chronic liver injury is of paramount importance.

In recent years, it has become clear that programmed cell death was not restricted to apoptosis, but comprised other forms of regulated cell death 6. Necroptosis is one of them, combining the molecular machinery of the extrinsic apoptotic pathways with an execution similar to necrosis 6, 7. Unlike apoptosis, which requires the activation of aspartate-specific proteases known as caspases, necroptosis is first driven by the activation of the receptor-interacting protein kinase (RIPK) 1 and 3, followed by the activation of the pseudo kinase mixed lineage kinase domain-like (MLKL) 7. Phosphorylation of MLKL leads to its oligomerization. Oligomerized MLKL then binds to and disrupts the plasma membrane releasing cellular components including the damage-associated molecular patterns (DAMPs), which exacerbate the inflammatory process 7. Necroptosis has emerged as a novel mode of cell death in various chronic liver diseases such as non-alcoholic steatohepatitis (NASH) 6, 8-11. Deficiency of *Mlkl* alleviates hepatic insulin resistance and glucose intolerance 12 and has a protective effect on NASH induced by high fat, fructose, and cholesterol diet (FFC) through inhibition of hepatocyte autophagy in hepatocytes 13. Pharmacological inhibition of necroptosis reduces hepatic inflammation and fibrosis in various murine models of liver diseases 12, 14. Although necroptosis was shown to play an important role in a number of liver diseases, the function of MLKL in liver fibrosis is still unclear. Moreover, there is growing evidence to indicate that MLKL function is not restricted to necroptosis but can also serve as a regulator of many diseases *via* non-necroptotic functions 15.

In the study from the group of Xin Xie published in the previous issues of *Theranostics* 16, the authors examined the role of MLKL in CCl_4_- and bile duct ligation (BDL)-induced liver injury and fibrosis (**Figure 1**). They showed that MLKL content positively correlated with a number of fibrotic markers in liver samples from both patients with and animal models of liver fibrosis. *Mlkl* deficiency in mice significantly reduced CCl_4_- and BDL-induced liver injury and fibrosis. Considering that hepatocyte injury is the main trigger of liver fibrosis, an adeno-associated virus (AAV) type 8 carrying *Mlkl* shRNA was used to specifically knock down *Mlkl* in hepatocytes. The authors demonstrated that AAV8-mediated specific knock down of *Mlkl* in hepatocytes remarkably alleviated CCl_4_-induced liver injury. The authors also showed that fibrosis reduction was not only due to the reduction in hepatocyte necroptosis but also to a reduction of HSCs activation, suggesting that targeting MLKL may be an effective way to treat liver fibrosis by acting both on the initiation (*i.e.,* death of hepatocytes) and progression (*i.e.,* HSC activation) stages of fibrogenesis.

Given the assumption that hepatocyte death is the key trigger of liver disease progression towards fibrosis, and the privileged role of MLKL in regulating necroptosis, it is not surprising that *Mlkl* deficiency protects mice from CCl_4_- and BDL-induced liver injury and fibrosis. However, *Mlkl* deficiency also reduced the activation of HSCs *in vitro*, possibly *via* the regulation of TGFβ/Smad 2/3 signaling pathway. It has been demonstrated that fully activated HSCs are resistant to apoptosis and can survive to prolonged serum deprivation, exposure to Fas ligand, NGF, TNF‐α, doxorubicin, etoposide, and oxidative stress mediators such as hydrogen peroxide, superoxide anion, and 4‐hydroxynonenal 17, 18. This resistance has been attributed to the antiapoptotic gene *Bcl-2*, which was overexpressed in HSCs in areas localized near fibrotic septa 18. Therefore, it would be interesting to evaluate if HSCs are also resistant to necroptosis. Moreover, since apoptosis and necroptosis are interconnected with each other but generally do not co-exist 19, it is possible that the acquired resistance of HSCs to apoptosis has facilitated the activation of pro-fibrogenic signaling pathways controlled by MLKL, especially if necroptosis was inhibited. The use of a conditional lecithin-retinol acyltransferase (Lrat)-cre/loxP based knockdown approach of MLKL in HSCs will be necessary to confirm these assumptions 20.

Cytoplasmic MLKL is translocated into the plasma membrane for necroptosis induction. However, preceding necroptosis induction, MLKL is also located to the nucleus 21. Three-dimensional analysis of immunocytochemistry has revealed that MLKL is located within the nucleus without being associated to the nuclear membrane 21. Noticeably, the necroptotic function of MLKL is independent of its translocation, and pharmacological inhibition of necroptosis by necrosulfonamide (NSA) has no effect on its nuclear translocation 21. Therefore, these data suggest that MLKL may have a necroptotic-independent function in regulating transcriptional activities. From this point of view, a recent study has revealed that MLKL interacts with RNA-binding motif protein 6 (RBM6) to promote the expression of several adhesion molecules including intercellular adhesion molecule-1 (ICAM-1), vascular cell adhesion molecule-1 (VCAM-1), and E-selectin 22. Moreover, *MLKL* deficiency compromises the invasion of the nasopharyngeal carcinoma cells by reverting epithelial-mesenchymal transition (EMT) 23. Given that MLKL appears to regulate adhesion molecules and EMT in cancer cells, we may hypothesize that MLKL exerts its necroptotic-independent function through transcriptional regulation by promoting EMT-like mechanisms 24 and thus HSCs trans-differentiation into scar-forming myofibroblasts.

While activation of MLKL has been reported to induce macrophage necroptosis, and targeting macrophage necroptosis may have therapeutic and diagnostic value in atherosclerosis 25, the data of Ren Guo *et al.* indicate that *Mlkl* deficiency does not influence bone marrow-derived macrophages (BMDM) polarization and cytokines production. Previous reports exploring the role of necroptosis in cerebral ischemia have shown that defective necroptosis induced polarization of macrophages/microglia toward M2 phenotype, but had not impact on polarization of naïve blood macrophages 26. Taken together, these results indicate that MLKL may have cell type-specific functions, not only related to cell fate but also to its embryonic origin.

Next to its role as an effector of necroptosis, MLKL also participates in the assembly of the NLRP3 (NOD-like receptor family, pyrin domain containing 3) inflammasome 27. MLKL-induced NLRP3 inflammasome formation and IL-1β cleavage occur before cell lysis. Furthermore, necroptotic activation of NLRP3, but not necroptotic cell death alone, is necessary for the activation of NF-κB in healthy bystander cells, suggesting that NLRP3 inflammasome activity is a driving force of inflammation in MLKL-dependent diseases 27. Therefore, it would be useful to evaluate the contribution of MLKL-dependent inflammasome in the progression of liver fibrosis.

The evidence presented by Ren Guo *et al.* contributes to a better understanding of the role of MLKL in the development of liver fibrosis. It clearly confirmed that MLKL does not only serve as an effector of necroptosis but exhibits non-necroptotic functions that should be considered in future drug therapy. Nevertheless, further studies are needed to evaluate the differential contribution of MLKL in non-parenchymal cells *vs.* hepatocytes and whether pharmacological inhibition can hold promise as a therapy for liver fibrosis.

## Figures and Tables

**Figure 1:**
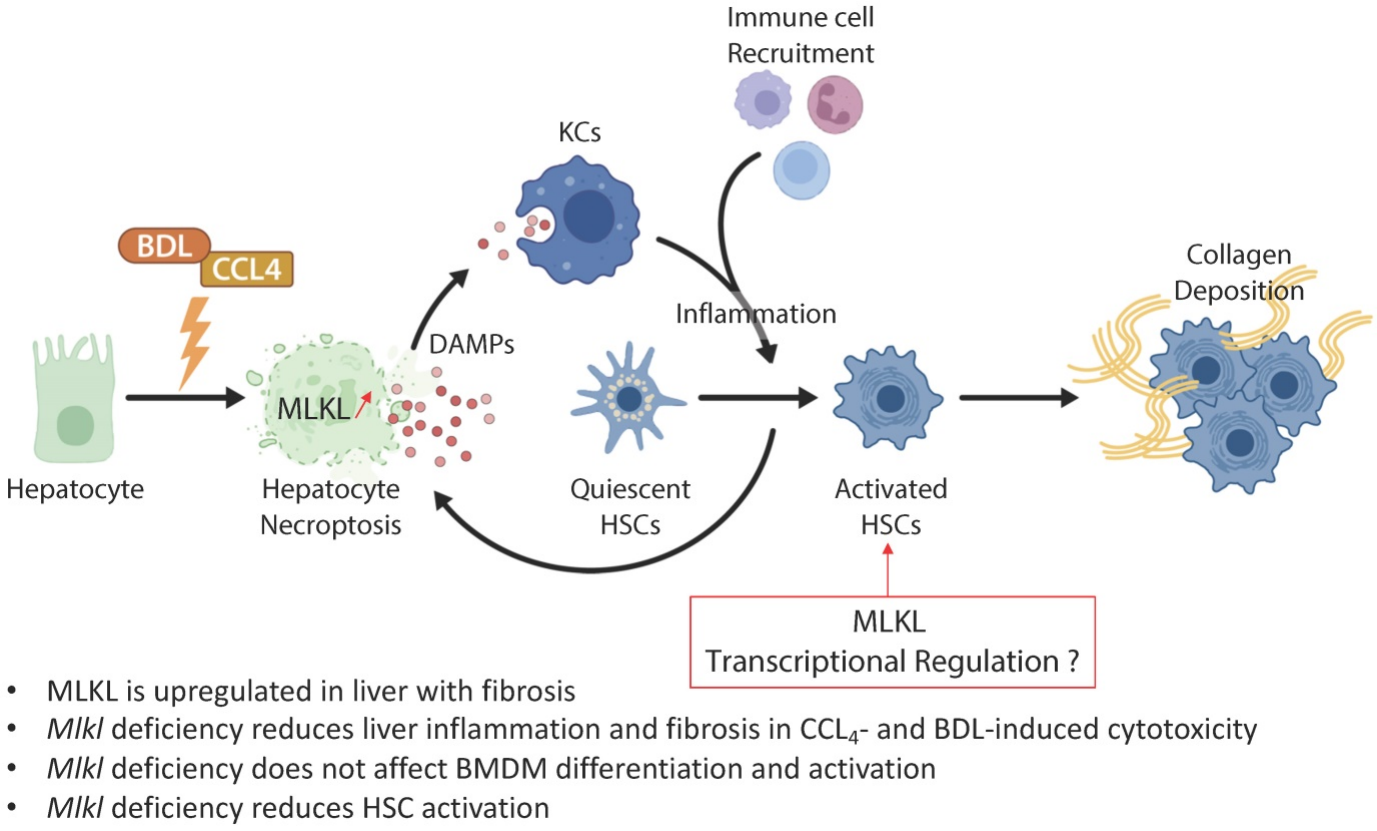
** MLKL contributes to liver inflammation and fibrosis in CCL4- and BDL-induced cytotoxicity.** Chronic hepatocyte injury causes hepatocyte necroptosis, which relies on MLKL. The release of DAMPs activates KCs, which secrete pro-inflammatory cytokines and thus enhance hepatocyte injury. Inflammation drives HSCs trans-differentiation into myofibroblasts (activated HSCs) that are ultimately responsible for the excessive synthesis, deposition and remodeling of extracellular matrix proteins in fibrosis. MLKL has multidirectional functions by promoting cell death in hepatocytes but also by favoring activation of HSCs possibly by transcriptional regulation. Abbreviations: DAMPs: damage-associated patterns; HSCs: Hepatic stellate cells; KCs: Kupffer cells; MLKL: Mixed lineage kinase domain-like protein.
